# The immune modulatory effects of mitochondrial transplantation on cecal slurry model in rat

**DOI:** 10.1186/s13054-020-03436-x

**Published:** 2021-01-07

**Authors:** Jung Wook Hwang, Min Ji Lee, Tae Nyoung Chung, Han A. Reum Lee, Jung Ho Lee, Seo Yoon Choi, Ye Jin Park, Chul Hee Kim, Isom Jin, Seong Hoon Kim, Hyo-Bum Kwak, Jun-Won Heo, Kwangmin Na, Sangchun Choi, Yong-Soo Choi, Kyuseok Kim

**Affiliations:** 1 Department of Biotechnology, CHA University, Gyeonggi-Do, South Korea; 2 Department of Emergency Medicine, CHA University School of Medicine, Gyeonggi-Do, South Korea; 3 Department of Emergency Medicine, CHA Bundang Medical Center CHA University, Gyeonggi-Do, South Korea; 4 Paean Biotechnology Inc., Seoul, South Korea; 5 Department of Biomedical Science, Program in Biomedical Science and Engineering, Inha University, Incheon, South Korea; 6 Department of Emergency Medicine, Ajou University School of Medicine, Suwon, South Korea

**Keywords:** Sepsis, Immune modulation, Hyperinflammation, Immune paralysis, Mitochondria transplantation, Mitochondria dysfunction

## Abstract

**Background:**

Sepsis has a high mortality rate, but no specific drug has been proven effective, prompting the development of new drugs. Immunologically, sepsis can involve hyperinflammation, immune paralysis, or both, which might pose challenges during drug development. Recently, mitochondrial transplantation has emerged as a treatment modality for various diseases involving mitochondrial dysfunction, but it has never been tested for sepsis.

**Methods:**

We isolated mitochondria from L6 muscle cells and umbilical cord mesenchymal stem cells and tested the quality of the isolated mitochondria. We conducted both in vivo and in vitro sepsis studies. We investigated the effects of intravenous mitochondrial transplantation on cecal slurry model in rats in terms of survival rate, bacterial clearance rate, and the immune response. Furthermore, we observed the effects of mitochondrial transplantation on the immune reaction regarding both hyperinflammation and immune paralysis. To do this, we studied early- and late-phase cytokine production in spleens from cecal slurry model in rats. We also used a lipopolysaccharide (LPS)-stimulated human PBMC monocyte model to confirm the immunological effects of mitochondrial transplantation. Apoptosis and the intrinsic apoptotic pathway were investigated in septic spleens.

**Results:**

Mitochondrial transplantation improved survival and bacterial clearance. It also mitigated mitochondrial dysfunction and apoptosis in septic spleens and attenuated both hyperinflammation and immune paralysis in the spleens of cecal slurry model in rats. This effect was confirmed with an LPS-stimulated human PBMC study.

**Conclusions:**

In rat polymicrobial cecal slurry model, the outcome is improved by mitochondrial transplantation, which might have an immunomodulatory effect.

## Background

Sepsis is defined as a life-threatening organ dysfunction caused by dysregulated host responses to infection [1]. Worldwide, it has a high incidence and mortality and continues to be a public health problem [2, 3]. Given this background, the WHO has announced sepsis as a global health priority [4]. There have been many attempts to develop drugs for sepsis, but none of the potential drugs have shown clinical efficacy.

Mitochondria are key components in cellular metabolism, cell growth, apoptosis, calcium homeostasis, redox status, etc., and their dysfunction has been implicated as a therapeutic target for various diseases [5]. Mitochondrial dysfunction has also been investigated as an important pathophysiological mechanism of sepsis development [6, 7]. Therapeutic drugs targeting mitochondrial dysfunction have been developed and tested in both preclinical and clinical studies [8]. However, no specific drugs are used in practice.

Mitochondrial transplantation has been proposed to be useful for the treatment of ischemia–reperfusion injury of the heart, and it shows promising clinical benefits with regard to neonatal congenital heart diseases [9, 10]. Mitochondrial transplantation enhances oxygen consumption, ATP synthesis, cell viability and postinfarct cardiac function [10]. Given these interesting findings, many diseases are being investigated as candidates for mitochondrial transplantation in both preclinical and clinical studies [11], but there have been no studies related to sepsis.

**Fig. 1:**
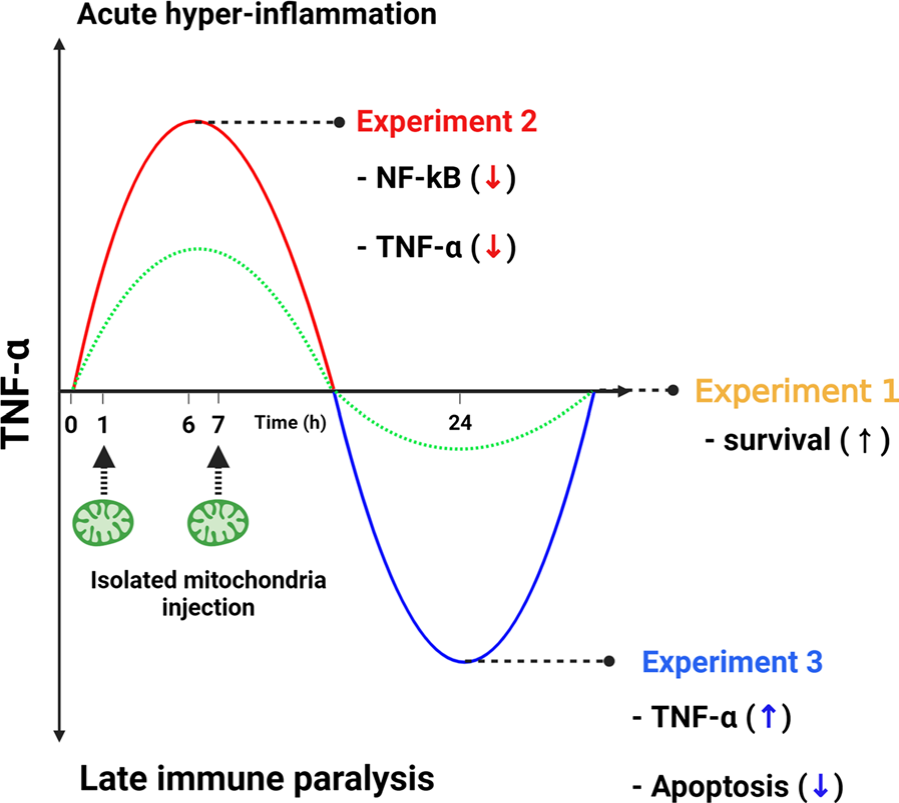
Overview of the effects of mitochondrial transplantation on sepsis and scheme of the experiments. Schematics showing experiment 1, in which mitochondrial transplantation increased the survival rate during sepsis; experiment 2, in which acute hyperinflammation was attenuated by mitochondrial transplantation; and experiment 3, in which mitochondrial transplantation decreased sepsis-induced apoptosis and attenuated late immune paralysis

Considering the importance of mitochondrial dysfunction in sepsis and the effects of transplanted mitochondria, we hypothesized that mitochondrial transplantation would have therapeutic potential for sepsis. To test this hypothesis, we used both in vivo and in vitro sepsis models. We investigated survival gains and immunomodulatory effects upon mitochondrial transplantation (Fig. 1). We also assessed the antiapoptotic and antifission effects of transplanted mitochondria.

## Methods

### Mitochondrial function assessment after induction of cecal slurry model in rats

We investigated changes in mitochondrial function in various tissues of cecal slurry model in rats. We measured the respiratory control ratio (RCR) with a high-resolution respirometer (Oroboros Oxygraph-2K, Oroboros Instruments, Innsbruck, Austria). The baseline RCR was measured in the liver, kidneys, spleen, and muscle, and then a cecal slurry was used to induce sepsis. To determine the baseline RCR, a small piece of each tissue was collected by biopsy. We repeated measurement of RCR in the same animals 24 h after sepsis induction. We compared the RCR values in animals with and without induction of sepsis.

### Mitochondria isolation

We isolated mitochondria using a previously described method with some modifications [12]. In brief, L6 cells (ATCC; CRL-1458, Manassas, VA, USA) were homogenized using a 26 G syringe in SHE buffer (0.25 M sucrose, 20 mM HEPES [pH 7.4], 2 mM EGTA, 10 mM KCl, 1.5 mM MgCl_2_ and 0.1% defatted bovine serum albumin [BSA] with 1 × protease inhibitor) and centrifuged at 1500×*g* for 5 min at 4 °C. The supernatant was centrifuged at 20,000×*g* for 10 min at 4 °C to obtain mitochondria. To isolate mitochondria from umbilical cord mesenchymal stem cells (UC-MSCs; IRB No. 201806-BR-029-03), UC-MSCs were homogenized using a 26 G syringe in SHE buffer and centrifuged at 1100×*g* for 3 min at 4 °C. The supernatant was centrifuged at 12,000×*g* for 15 min at 4 °C. The pellet was resuspended using SHE buffer without BSA, and the suspension was centrifuged at 20,000×*g* for 10 min at 4 °C to obtain mitochondria. For all experiments, the isolated mitochondria were stored at 4 °C until use.

### Quality control of isolated mitochondria

The sizes and zeta potentials of the isolated mitochondria were measured using a Malvern Nano-ZS 90 laser analyzer (Malvern Instruments, Malvern, UK), and the mitochondrial concentrations were analyzed with a Pierce BCA protein assay kit (Thermo Fisher, Waltham, MA, USA). Purity of isolated mitochondria from stained L6 cells by Mitotracker Red (Molecular Probes, Eugene, OR, USA) were analyzed using CytoFLEX software (Beckman Coulter, California, USA). Other organelles in isolated mitochondria were detected using Western blot analysis.

### In vivo cecal slurry model in rats

Sprague–Dawley rats weighing 270–330 g were used. The rats were housed in a controlled environment with access to standard food and water ad libitum for 7 days before the experiment.

**Fig. 2:**
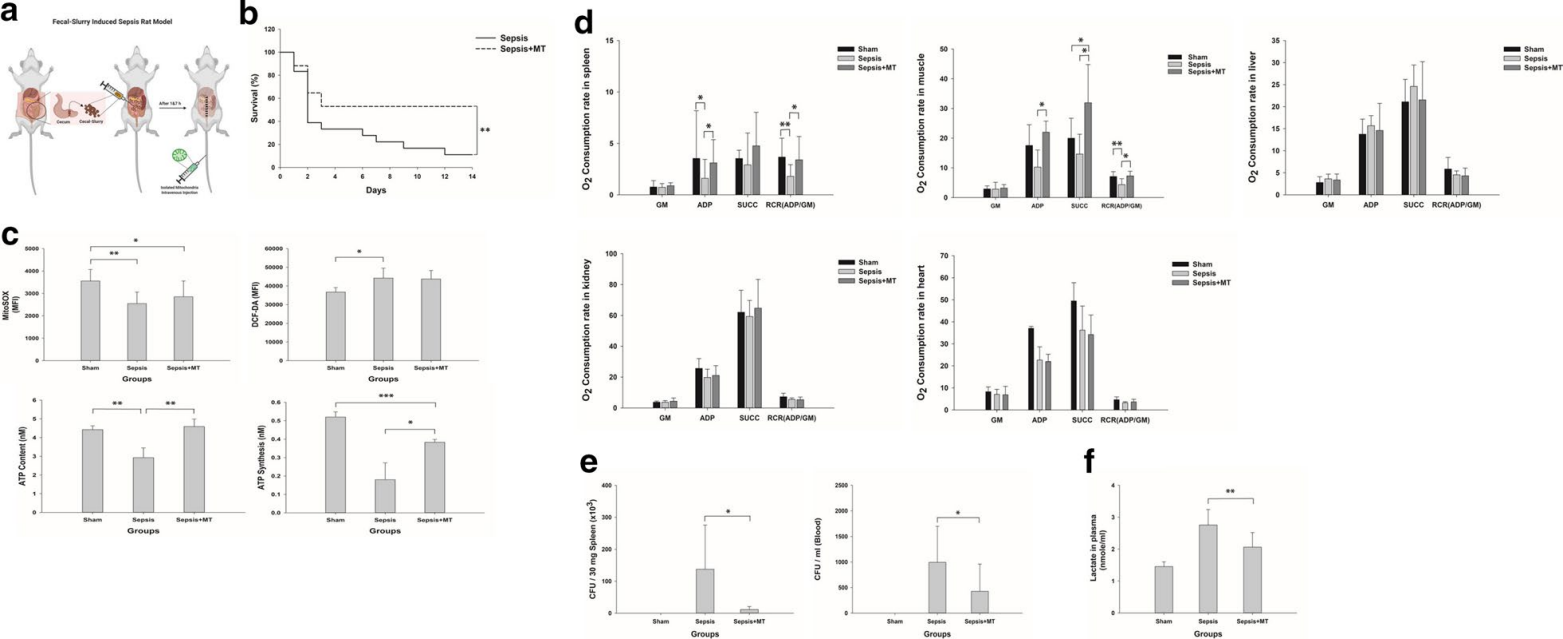
Effects of mitochondrial transplantation on sepsis in animal studies. **a** Conceptual figure of the rat sepsis model. **b** Kaplan–Meier survival plots of the studied animals (*n* = 16). **p < 0.01 and *p < 0.05 compared with the sepsis group. **c** The MFIs of MitoSOX and DCF-DA were measured in the spleen 24 h after induction of sepsis (top). Sham group, *n* = 3; sepsis and sepsis + MT mice, *n* = 9 per group). **p < 0.01 and *p < 0.05 compared with the sham group. ATP content and synthesis were measured in the spleen 24 h after induction of sepsis (bottom). Sham group, *n* = 3; sepsis and sepsis + MT group, *n* = 3 to 4 per group. ***p < 0.01 compared with the sham group and **p < 0.01 and *p < 0.05 compared with the sham and sepsis groups, respectively. **d** Oxygen consumption trace in muscle, spleen, liver, kidney and heart tissues. Spleen, *n* = 6 to 14 per group; muscle, *n* = 4 to 12 per group; liver, *n* = 10 to 13 per group; kidneys, *n* = 3 to 6 per group; heart, *n* = 2 to 6 per group. **p < 0.01 and *p < 0.05 compared with the sepsis group. **e** CFUs were counted in spleen and blood 24 h after induction of sepsis. Spleen, *n* = 5 to 14 per group; blood, *n* = 8 per group. *p < 0.05 compared with the sepsis group. **f** Lactate concentrations were assessed in plasma 24 h after induction of sepsis. Plasma, *n* = 10 per group. **p < 0.01 compared with the sepsis group. *MT* mitochondria, *CFU* colony-forming unit

We used a body weight-adjusted polymicrobial sepsis model according to a previous study [13]. In brief, we used inhalation anesthesia with isoflurane for the short term and then injected intramuscular Zoletil (50 mg/kg) and Xylazine (10 mg/kg) before experiments. Feces was collected from donor rats. The collected feces was diluted with 5% dextrose saline at a ratio of 1:3. This fecal slurry was vortexed to make a homogeneous suspension before administration into the intraperitoneal cavity. The volume of cecal slurry given to each animal was adjusted on the basis of the body weight of the recipient rat. We injected ceftriaxone (150 mg/kg) intramuscularly and performed subcutaneous fluid resuscitation (30 mL/kg 5% dextrose saline). Thereafter, the rats were randomly assigned to the mitochondrial transplantation and control groups (Fig. 2a).

*Experiment 1* Survival study. In the survival study, we introduced severe cecal slurry model with 6.0 mL/kg cecal slurry. Mitochondria were administered 1 h and 7 h after sepsis induction at a dose of 50 µg via the tail vein. Survival was monitored every 12 h for 14 days.

*Experiment 2* Acute hyperinflammation study. In this study, 6.0 mL/kg cecal slurry was administered, and blood and tissues were harvested 6 h after sepsis induction. We measured TNF-α, IL-6, and IL-10 in the blood and spleen. Plasma lactate was also measured.

*Experiment 3* Late immune paralysis study. In the immune paralysis study, mild cecal slurry model of 4.5 mL/kg cecal slurry was administered, and blood and tissues were harvested 24 h after sepsis induction. We investigated mitochondrial function using high resolution respirometry in the spleen, liver, kidneys, muscle, and heart. We measured IL-6, IL-10 and lactate in the blood and spleen. TUNEL staining was performed to observe apoptosis in the spleen. Splenocytes were isolated for an ex vivo lipopolysaccharide (LPS) response study. The colony-forming units (CFUs) in the blood and spleen were counted. In vitro phagocytic activity of peripheral blood mononuclear cell (PBMC) was determined.

### Assessment of intracellular reactive oxygen species (ROS) and mitochondrial ROS levels in the spleen

ROS levels were analyzed according to the reagent manufacturer’s instructions. Isolated splenocytes were treated with 10 µM DCF-DA (Invitrogen, Carlsbad, CA, USA) or 5 µM MitoSOX (Invitrogen, Carlsbad, CA, USA) at 37 °C for 30 min in the dark and then washed twice with DPBS. The ROS population was gated, and a histogram was created. The mean fluorescence intensity (MFI) was obtained using CytoFLEX software (Beckman Coulter, California, USA).

### ATP assay

ATP content and ATP synthesis capacity were measured by CellTiter-Glo Luminescent Cell Viability Assay (Promega, Madison, WI, USA). To determine ATP synthesis capacity, 1 × 10^5^ isolated splenocytes were placed into white 96-well plates, 5 mM ADP solution was added, and the splenocytes were incubated for 45 min at 37 °C. To measure ATP content, 1 × 10^5^ isolated splenocytes were placed into 96-well white plates. In both experiments, 100 μL of CellTiter-Glo reagent was added to each well; the plates were then gently shaken for 2 min, and the reaction was allowed to proceed for 10 min at room temperature in the dark. The plates were read with a luminometer (Synergy HTX, BioTex, Winooski, VT, USA).

### CFU assay

Blood and spleen samples were used to count bacterial CFUs in cecal slurry model in rats. Thirty milligrams of spleen tissue was homogenized in 500 μL of DPBS and centrifuged at 12,000 rpm for 10 min at 4 °C. The cell pellets were resuspended in 1 mL of DPBS (to form stock samples). The stock samples were diluted 1:10 with DPBS. To count blood CFUs, 800 μL of DPBS was added to 200 μL of blood. The samples were spread on TSA agar plates (BD Biosciences) without antibiotics and incubated at 37 °C overnight, and the bacterial colonies were counted for analysis.

### Lactate assay

Lactate concentrations in plasma were evaluated with a Lactate Colorimetric Assay Kit (BioVison, Milpitas, CA, USA). Briefly, sample and reaction mix buffer were added to the wells and then incubated for 30 min at room temperature. After 30 min, the optical density of each well was detected at 450 nm by a microplate reader (VersaMax with SoftMax Pro software, Molecular Devices, CA, USA).

### Apoptosis study

TUNEL staining of spleen: Apoptosis was assessed by the terminal deoxynucleotidyl transferase-mediated dUTP nick-end labeling (TUNEL) method. A DeadEnd Colorimetric TUNEL system (Cat. No G7130, Promega, South Korea) was used to perform TUNEL staining on paraffin-embedded sections of 3 μm thickness according to the manufacturer’s instructions. The tissue sections were incubated with biotinylated nucleotides, and streptavidin conjugated with horseradish peroxidase (HRP) was used for detection. Finally, color development was performed using a diaminobenzidine substrate. The slides were examined under a microscope (Nikon, Tokyo, Japan) at 400× magnification. The TUNEL-positive cells were counted in five non-successive fields per sample. The apoptotic index (AI) was defined as the number of apoptotic cells per white pulp sample. Intrinsic apoptotic pathway assessment: BAX, BCL-2, cytochrome c, cleaved caspase 9, caspase 9, and cleaved caspase 3 levels were measured using Western blot analysis.

### Mitochondrial dynamics study

Mitochondrial fusion and fission were evaluated using OPA1, mitofusin-2, DRP-1 and pDRP-1 (Ser616).

### Ex vivo splenocyte stimulation with LPS

Spleens were harvested 24 h after sepsis induction (in vivo experiment 3), and splenocytes were isolated and stimulated with LPS to see and compare the immune paralysis. TNF-α, IL-6, and IL-10 were measured 5 and 24 h after LPS stimulation. In brief, isolated splenocytes were seeded in 6-well plates at 5 × 10^5^ cells/mL in RPMI1640 with 10% fetal bovine serum (FBS; Gibco, Paisley, UK), and 1 μg/mL LPS (Escherichia coli O111: B4; Sigma-Aldrich, St. Louis, MO, USA) was added to each well. After 5 h or 24 h, the culture medium was collected, and then TNF-α was analyzed using a TNF-α ELISA kit (R&D Systems Inc., Minneapolis, MN, USA).

### In vitro LPS stimulation model

Blood collection and cell separation: Thirty milliliters of peripheral venous blood was drawn from healthy volunteers (CHAMC 2019-07-040) into heparin-treated tubes, and peripheral blood mononuclear cells (PBMCs) were obtained by the Ficoll method. CD14 + monocytes were isolated from human PBMCs using CD14 Microbeads, an LS column, and a QuadroMACS Separator (Miltenyi Biotec, Auburn, CA, USA). Isolated monocytes were cultured in RPMI-1640 medium (Gibco) supplemented with 10% fetal bovine serum, penicillin–streptomycin (Gibco), and 0.05 mM beta-mercaptoethanol (Gibco). The standard cell concentration was 1 × 10^6^ cells/mL.

**Fig. 3:**
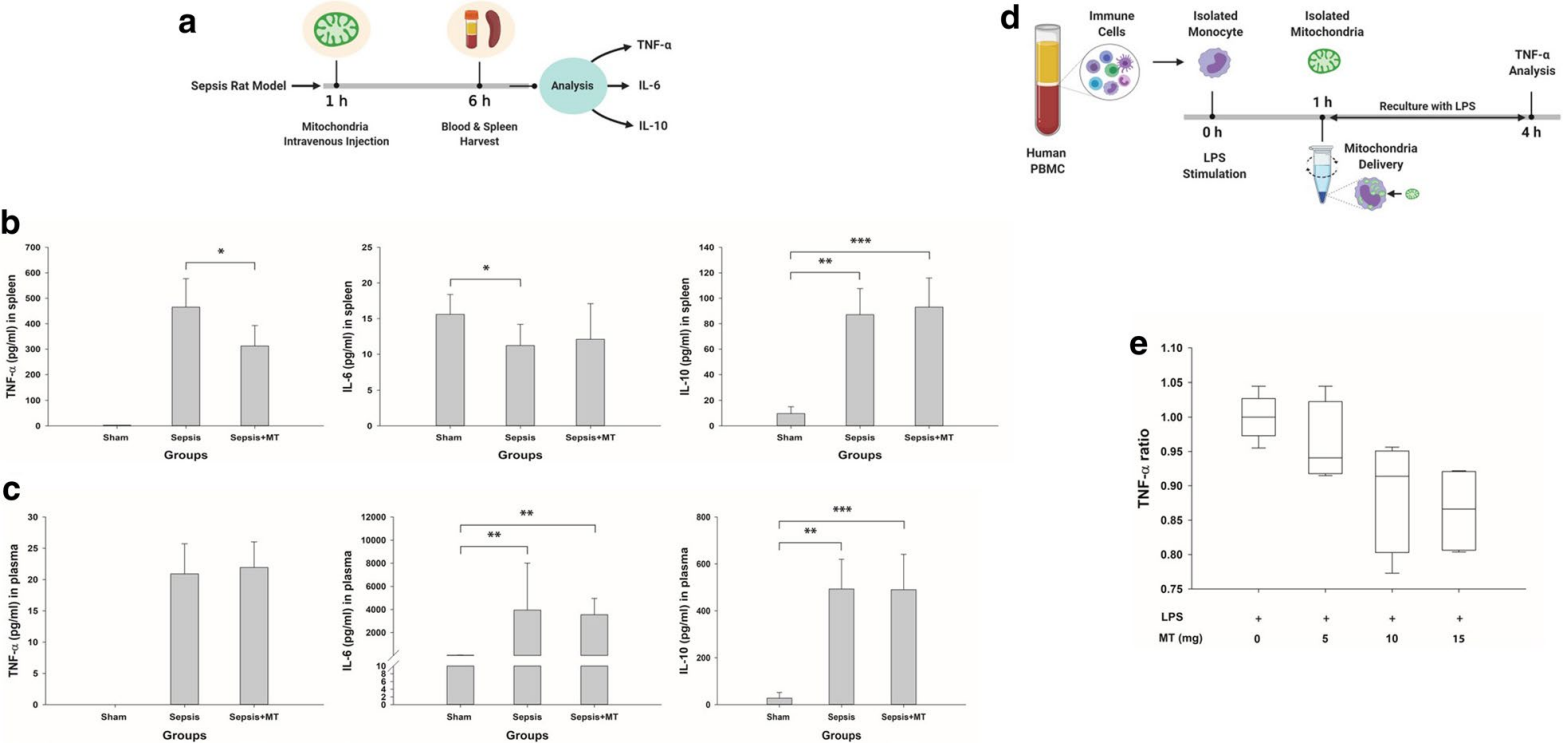
Effects of mitochondrial transplantation on the acute hyperinflammatory stage of sepsis. **a** Flow diagram of the experimental procedures used to assess the immunomodulatory effects of mitochondrial transplantation in an in vivo sepsis model in the hyperinflammation stage. **b** The levels of the cytokines TNF-α, IL-6 and IL-10 were measured in the spleen 6 h after induction of sepsis. Sham mice, *n* = 4; sepsis and sepsis + MT mice, *n* = 6 per group. ***p < 0 .001, **p < 0 .01 and *p < 0.05 compared with the sham or sepsis group. **c** The levels of the cytokines TNF-α, IL-6 and IL-10 were measured in the plasma 6 h after induction of sepsis. Sham mice, *n* = 4; sepsis and sepsis + MT mice, *n* = 6 per group. ***p < 0 .001 and **p < 0 .01 compared with the sham or sepsis group. **d** Flow diagram of the experimental procedures used to assess the immunomodulatory effects of mitochondrial transplantation in an in vitro sepsis model in the hyperinflammation stage. **e** Isolated monocytes from human PBMCs were stimulated with LPS and then delivered isolated mitochondria. The supernatants were collected, and TNF-α expression was measured (*n* = 4 per group). *MT* mitochondria, *PBMC* peripheral blood mononuclear cell, *LPS* lipopolysaccharide

Hyperinflammation model (Fig. 3d): Cells were stimulated with 10 ng/mL LPS for 4 h, and the supernatants were stored at − 70 °C for TNF-α analysis. In the mitochondrial transplantation group, isolated mitochondria were mixed with cells 1 h after LPS exposure, and the mixtures were centrifuged at 1500×*g* for 5 min at 4 °C. The cells were washed twice with ice-cold PBS and resuspended in fresh medium containing 10 ng/mL LPS. In the control group, the same protocol was performed, including centrifugation, without mitochondrial transplantation.

**Fig. 4:**
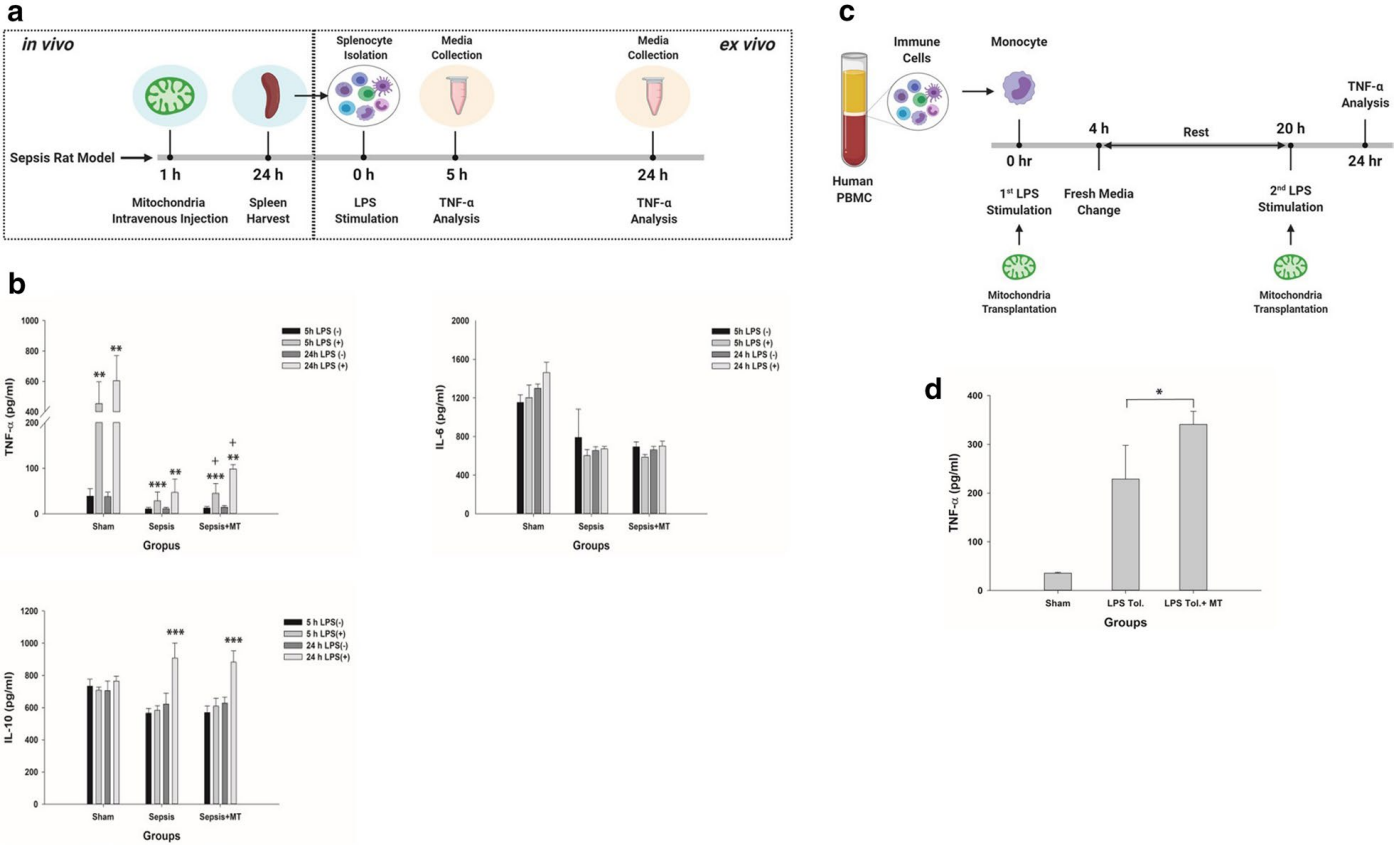
Effects of mitochondrial transplantation on the late immunosuppressive stage of sepsis. **a** Flow diagram of the experimental procedures used to assess the immunomodulatory effects of mitochondria on an ex vivo sepsis model in the immunosuppressive stage. **b** Splenocytes from rat spleens were stimulated with LPS, and then the supernatants were collected. The levels of the cytokines TNF-α, IL-6 and IL-10 were measured after 5 and 24 h, respectively. Sham, *n* = 6 per group; sepsis and sepsis + MT, *n* = 10 to 13 per group. ***p < 0 .001 and **p < 0 .01 compared with the no-LPS (-) group. ^†^*p* < 0.05 compared with the sepsis group. **c** Flow diagram of the experimental procedures used to assess the immunomodulatory effects of mitochondria on an in vitro sepsis model in the immunosuppressive stage. **d** Isolated monocytes from human PBMCs were stimulated with LPS and then treated with isolated mitochondria. The supernatants were collected, and TNF-α expression was measured (*n* = 4 per group). *p < 0.05 compared with the LPS Tol. group. *MT* mitochondria, *PBMC* peripheral blood mononuclear cell, *LPS* lipopolysaccharide, *Tol.* tolerance

Immune paralysis model (Fig. 4c): This model, also known as the endotoxin tolerance model, was induced by stimulation with 10 ng/mL LPS for 4 h. The LPS was then washed away using fresh RPMI medium. The cells were allowed to rest for 16 h and were subsequently restimulated with 10 ng/mL LPS for 4 h. Mitochondria were transplanted as described above during each LPS stimulation. TNF-α was measured after the second LPS stimulation.

In both hyperinflammation and immune paralysis model, we tested the effect of electron transfer chain inhibitors on immune-modulatory effect of mitochondrial transplantation. We incubated isolated mitochondria with metformin (complex I inhibitor) or oligomycin (complex V inhibitor). In brief, isolated mitochondria from UC-MSCs were treated 10 mM metformin and 1 μM oligomycin, and then reacted 37 °C for 30 min. After reaction, it was washed three times using DPBS and used in the experiment.

### Macrophage polarization

In hyperinflammation model, we tested the effect of macrophage polarization on mitochondrial transplantation. In brief, isolated monocyte from human PBMC were seeded in 24-well plates at 2 × 10^5^ cells/mL in RPMI1640 with 10% fetal bovine serum (FBS; Gibco, Paisley, UK), and 1 μg/mL LPS (Escherichia coli O111: B4; Sigma-Aldrich, St. Louis, MO, USA) was added to each well with or without mitochondria. After 6 h, monocytes were stained with FITC-CD80 Ab for M1 macrophage (eBioscience, San Diego, CA, USA) and analyzed using CytoFLEX software.

### Phagocytosis study

Phagocytosis was evaluated in immune paralysis model. In in vitro model of immune paralysis, phagocytic activity of isolated monocyte from human PBMC was investigated. Briefly, isolated monocytes were seeded in 24-well plates at 2 × 10^5^ cells/mL in RPMI1640 with 10% fetal bovine serum, and 1 μg/mL LPS were added twice to each well with or without mitochondria. After 24 h, monocyte was incubated with *E. coli* labeled by fluorescein (FITC). Bacteria ingested by phagocytes were quantified by flow cytometry [14–16].

### Western blot analysis

Tissues of whole spleen and isolated mitochondria from L6 cells were lysed using RIPA buffer (Biosesang, Seongnam, Korea) with a protease inhibitor cocktail (Sigma-Aldrich). Twenty micrograms of protein was separated by 10 or 12% SDS-PAGE, and the separated proteins were transferred to polyvinylidene difluoride (PVDF) membranes (Millipore, Bedford, MA, USA). The membranes were blocked in 5% BSA for 1 h at room temperature and then incubated with primary antibodies overnight at 4 °C (Caspase 9 [1:1000, 9508S, Cell Signaling], BAX [1:1,000; sc-493, Santa Cruz], BCL-2 [1:1,000; sc-7382, Santa Cruz], Cytochrome c [1:1,000; sc-13156, Santa Cruz], Cleaved Caspase 3 [1:1,000, #9661S, Cell Signaling], β-actin [1:1,000; sc-47778, Santa Cruz], OPA-1 [1:000; 80471S, Cell Signaling]. DRP-1 [1:000; 8570S, Cell Signaling], p-DRP-1 (ser616) [1:1000; 3455S, Cell Signaling], PCNA [1:1000; sc-25280, Santa Cruz], KDEL [1:1000; sc-58774, Santa Cruz] and AIF [1:1000; sc-55519, Santa Cruz]). The membranes were washed with TBST, incubated with the appropriate HRP-conjugated anti-rabbit IgG (1:1,000; sc-2357, Santa Cruz) or anti-mouse IgG (1:1,000; sc-516102, Santa Cruz) secondary antibodies for 1 h at room temperature, and then washed with TBST six times. The protein expression on each membrane was visualized using ECL Plus reagent (Bio-Rad, Hercules, CA, USA) using a Luminescent Image Analyzer (LAS-1000, Fuji Film, Tokyo, Japan).

### Cytokine measurements

The levels of the cytokines IL-6 (R6000B, R&D Systems, MN, USA), IL-10 (ab214566, Abcam, MA, USA), and TNF-α (ab236712, Abcam, MA, USA) in spleen homogenates and plasma were measured using ELISA kits according to the manufacturer’s instructions. To assess cytokine levels in the spleen, frozen tissue was homogenized in RIPA buffer with protease inhibitor cocktail. The optical density at 450 nm was detected by a microplate reader (VersaMax with SoftMax Pro software, Molecular Devices, CA, USA).

### Analysis of the distribution of transplanted mitochondria after intravenous injection

Imaging analysis: Isolated mitochondria were stained with MitoTracker Red CMXROS (Molecular Probes, Eugene, OR, USA) and then washed with DPBS twice. The stained mitochondria were injected intravenously into cecal slurry model in rats. After 24 h, the spleens were soaked in optimum cutting temperature (OCT) compound, and then the samples were frozen in liquid nitrogen and stored at − 80 °C. Ten-micrometer-thick sections of the samples were cut using a Leica CM1950 cryostat (Leica Biosystems, Nussloch, Germany) and placed on coated glass slides. The slides were observed using fluorescence microscopy (ECLIPSE E400, Nikon, Tokyo, Japan).

### Statistical analysis

The Shapiro–Wilk test was performed to determine the normality of the data. Normally distributed data are presented as the mean ± standard deviation and were compared with independent *t* tests. If the data did not fit a normal distribution, they are presented as the median and interquartile range and were analyzed by Mann–Whitney *U* test or Kruskal–Wallis test. Survival rates were compared by Kaplan–Meier log-rank test. A *p* value of < 0.05 was defined as statistically significant. All analyses were performed with Sigma Plot software (SYSTAT Software, San Jose, CA).

## Results

### Mitochondrial function after induction of sepsis

Mitochondrial respiration after cecal slurry model in rats was significantly decreased in spleen and muscle tissues but not in liver or kidney tissues (Additional file 1: figure S1).

### Characteristics of isolated mitochondria

The membrane potential of the isolated mitochondria was − 15.6 ± 1.26 mV, and the size was 575 ± 145 nm (Additional file 2: figure S2A). The protein content of the isolated mitochondria from 2 × 10^7^ L6 cells was 377 ± 1.46 μg (Additional file 2: figure S2B). The ATP content of the isolated mitochondria was 17.5 ± 1.5 nM, and the synthesized ATP content after ADP treatment was 2604 ± 44.3 nM (Additional file 2: figure S2C). The purity of isolated mitochondria was 94.26% and other organelles was 4.18% (Additional file 2: figure S2D). Other organelles also existed in isolated mitochondria, confirmed with PCNA (Nucleus) and KDEL (ER) (Additional file 2: figure S2E).

### Distribution of transplanted mitochondria in septic spleens

The red fluorescence of MitoTracker Red-stained mitochondria was observed in septic spleen tissue (Additional file 3: figure S3).

### Mitochondrial respiration changes in septic spleens

Mitochondrial respiration as measured by RCR was significantly higher in spleen and muscle tissue with mitochondrial transplantation than in that without mitochondrial transplantation. However, there were no differences in the liver and kidneys (Fig. 2d, Additional file 4: figure S4). In addition, the ATP content and ATP synthetic capacity were significantly decreased in cecal slurry model in rats, but this effect was mitigated by mitochondrial transplantation (Fig. 2c bottom). Intracellular and mitochondrial ROS levels in the spleen were not significantly altered with mitochondrial transplantation (Fig. 2c top).

### Survival rate, bacterial clearance, and plasma lactate levels

The survival rate significantly increased with mitochondrial transplantation from 10 to 50% (Fig. 2b). Mitochondrial transplantation significantly reduced the bacterial burden in both the spleen and blood (Fig. 2e). The lactate level was decreased by mitochondrial transplantation (Fig. 2f).

### Immunomodulatory effects

In the hyperinflammation model of cecal slurry model in rats (6 h after sepsis induction) (Fig. 3a), mitochondrial transplantation attenuated inflammation, as shown by reduced TNF-α levels in the spleen (Fig. 3b). IL-6 and IL-10 levels in the plasma were not changed by mitochondrial transplantation (Fig. 3c). In the immune paralysis model (24 h after sepsis induction) (Fig. 4a), mitochondrial transplantation significantly increased TNF-α production in ex vivo splenocytes stimulated with LPS (Fig. 4b).

### In vitro LPS stimulation of human monocytes

In the hyperinflammation model (Fig. 3d), TNF-α production in monocytes was attenuated by mitochondrial transplantation (Fig. 3e). IL-6 was not changed by mitochondrial transplantation (Additional file 5: figure S5A) and IL-10 was not detected (data not shown). This effect was blunted by the pretreatment of metformin or oligomycin (Additional file 6: figure S6A, B). In the immune paralysis model (Fig. 4c), mitochondrial transplantation increased TNF-α production but not to the level of the sham group (Fig. 4d). IL-6 was not changed by mitochondrial transplantation (Additional file 5: figure S5B) and IL-10 was not detected (data not shown).

### Macrophage polarization

In hyperinflammation model, the macrophage polarization of monocytes from human PBMC was decreased in 1st LPS + 1st MT group compared to 1st LPS. (Additional file 7: figure S7).

### Phagocytic effects

In immune paralysis model with human monocyte, the phagocytosis of *E. coli* was increased in 2nd LPS + MT group compared to 2nd LPS. This was partially recovered with mitochondrial transplantation (Additional file 8: figure S8).

### Effects on apoptosis in the spleen

**Fig. 5:**
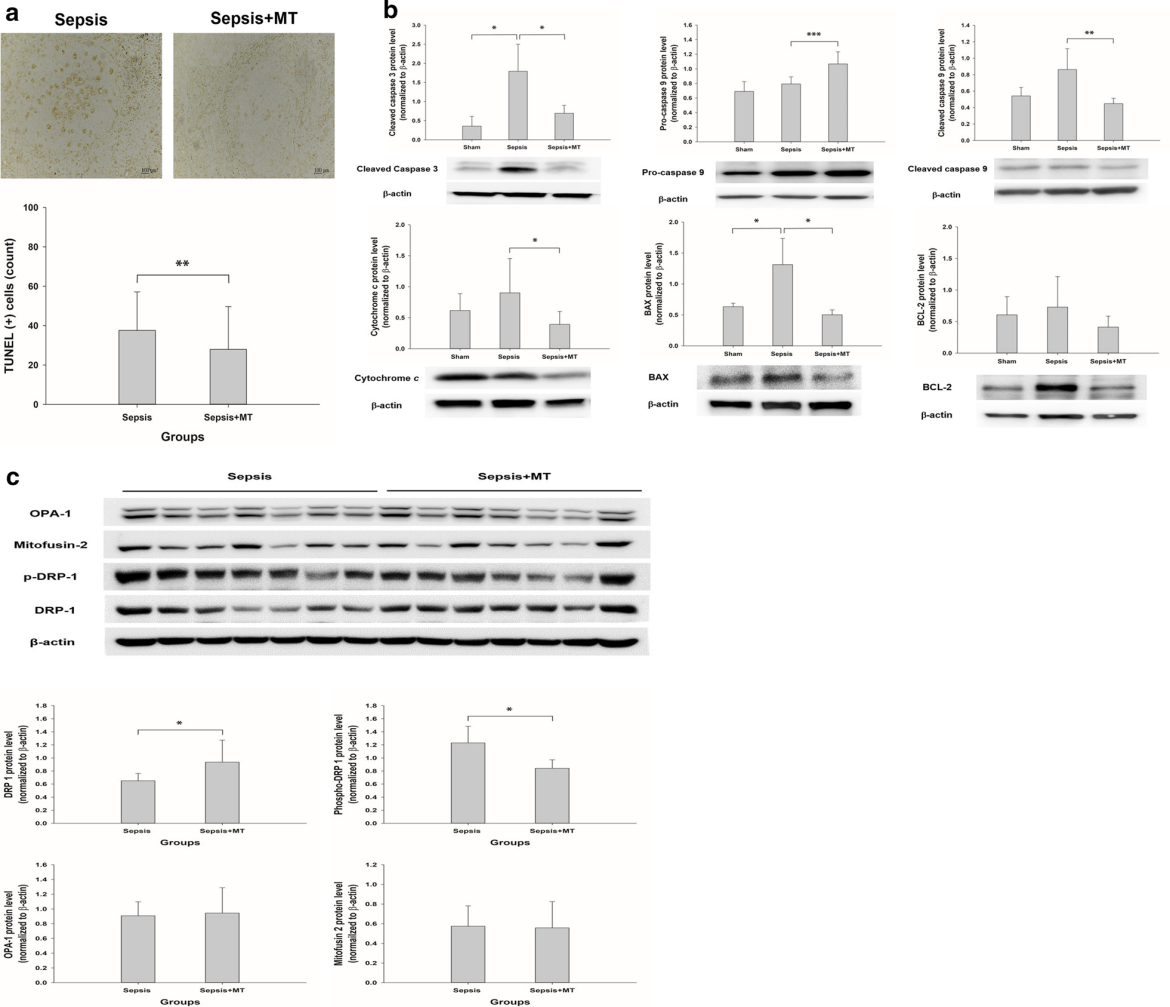
Effects of mitochondrial transplantation on apoptosis and mitochondrial fusion and fission. **a** TUNEL assay of the white pulp of the spleen (top). Scale bars, 100 μm. The numbers of apoptotic cells in the spleen were determined (bottom). **p < 0.01 compared with the sepsis group. **b** Intrinsic apoptosis markers in the spleen were measured by immunoblotting 24 h after induction of sepsis. Cleaved caspase 3, *n* = 4 per group; pro-caspase 9 and cleaved caspase 9, *n* = 3 to 8 per group; Cytochrome c, *n* = 4 to 9 group; BAX, *n* = 4 to 9 per group; BCL-2, *n* = 4 to 9 per group. ***p < 0 .001, **p < 0 .01 and *p < 0.05 compared with the sham or sepsis group. **c** Mitochondrial dynamics markers in the spleen were measured by immunoblotting 24 h after induction of sepsis (*n* = 7 per group for each marker). *p < 0.05 compared with the sepsis group. *MT* mitochondria, *TUNEL* terminal deoxynucleotidyl transferase dUTP nick end labeling

TUNEL staining showed that the average number of apoptotic cells in the sepsis group was 37.6; this number decreased significantly to 28 with mitochondrial transplantation (Fig. 5a). The protein expression of BAX, cleaved caspase 3, cleaved caspase 9 and cytochrome c was significantly attenuated by mitochondrial transplantation. In contrast, BCL-2 protein levels tended to be decreased in the mitochondrial transplantation group (Fig. 5b).

### Effects on mitochondrial dynamics

Mitochondrial fission was significantly decreased by mitochondrial transplantation, as shown by a decrease in p-DRP-1 levels (Fig. 5c).

## Discussion

This study is the first to demonstrate the effects of mitochondrial transplantation on sepsis. Clinical outcomes such as survival rate, bacterial clearance, and lactate levels were in favor of mitochondrial transplantation.

We found that mitochondrial transplantation attenuated apoptosis in the spleen, which might explain why it improved bacterial clearance. Immune cell apoptosis is considered a main factor contributing to immunosuppression in sepsis [17–19]. Consistent with this idea, we found that mitochondrial transplantation mitigated immune paralysis in the model of ex vivo splenocyte LPS stimulation (24 h after sepsis). This finding was confirmed through an in vitro human monocyte study with an endotoxin tolerance model. Therefore, the results might indicate that mitochondrial transplantation exerts an immune-enhancing effect on sepsis in the immune paralysis phase. Interestingly, both in vivo and in vitro human monocyte studies showed that in the hyperinflammatory acute phase of sepsis (6 h after sepsis induction), mitochondrial transplantation attenuated the hyperinflammatory reaction (TNF-α), indicating that it exerted an immunosuppressive effect. In this study, the mechanism underlying this phenomenon was not investigated. Recently, an intriguing hypothesis was proposed: that mitochondrial metabolism can be an immune system input and act as a signal to control the immune functions of a cell [20]. Mitochondrial transplantation can enhance oxidative phosphorylation (OXPHOS) in immune cells, which is characteristic of M2 macrophages or Tregs. M2 macrophages and Tregs dampen inflammatory responses by producing anti-inflammatory cytokines [21]. Our results regarding cytokine levels 6 h after the in vivo sepsis model and the in vitro LPS stimulation model might support this hypothesis. Also, the blunted effect of mitochondrial transplantation by ETC inhibitor shown in this study partly support this mechanism. Taken together, our findings indicate that mitochondrial transplantation has an immunosuppressive effect in the hyperinflammatory phase and an immune-enhancing effect in the late immune paralysis phase of sepsis. These results imply that mitochondrial transplantation has a bimodal immunomodulatory effect on sepsis. We might infer that the immune enhancing effects of mitochondrial transplantation could originate from the reduction of apoptosis of immune cells. On the contrary, the immune suppressive effect might be the result of shift from M1 to M2 polarization, which was partly supported by our experiment. Further study is needed to elucidate the mechanisms underlying this dual effect.

Traditionally, a hyperinflammatory systemic response, i.e., a cytokine storm, was considered to be the major immunological disorder in sepsis. In recent decades, however, this concept has changed; currently, immunosuppression or immune paralysis is considered to be the main contributor to mortality and morbidity [22–25]. Immune paralysis might either follow the initial hyperinflammatory reaction or occur concomitantly with it [26–28]. This might complicate sepsis treatment since it necessitates contradictory immune treatment. If septic patients exhibit hyperinflammation, they should be administered immunosuppressive drugs such as steroids or IL-1/IL-6 receptor antagonists. However, if the patients exhibit immune paralysis, they should be administered an immune-enhancing agent such as GM-CSF, INF-gamma, or a PD-1 antagonist. With this background, current clinical trials on sepsis treatments are utilizing biomarkers such as HLA-DR, PD-1, PD-L1, and serum ferritin to differentiate the immune statuses of enrolled patients. This approach sounds reasonable; however, considering the dynamic changes in immune status that occur during sepsis, diagnostic and therapeutic time gap might be problematic. In this respect, mitochondrial transplantation could be an intriguing treatment option because it has both anti-inflammatory and immune-enhancing effects, as shown in this study.

With regard to different disease models, mitochondrial transplantation has been shown to reduce apoptosis in a myocardial ischemia/reperfusion model [29]. Our study also showed an antiapoptotic effect of mitochondrial transplantation, and the intrinsic mitochondria-mediated pathway of apoptosis might be involved in this effect. Fission has been associated with cytochrome c release, and this could cause intrinsic apoptosis. In this study, fission was decreased with mitochondrial transplantation, which might attenuate apoptosis. We expected the higher level of BCL-2, but it is not significantly different between groups. Instead, the BCL-2 level was a little decreased in mitochondrial transplantation group. We did not know why this happened. However, not all studies about apoptosis showed increased level of BCL-2. Actually, BCL-2 prevents BAX/BAK oligomerization to decrease apoptosis, and in our study, BAK level was significantly lower in mitochondrial transplantation group, which might reduce the necessity for increased in BCL-2 [30, 31].

Mitochondrial dysfunction is well known to occur in the context of sepsis [6, 7]. In this study, we also investigated changes in mitochondrial function after induction of sepsis. Uniquely, we observed changes in RCR within the same animals using biopsy without sacrificing the animals. Interestingly, we found that mitochondrial function 24 h after sepsis was significantly decreased only in spleen and muscle tissues; it was not decreased in liver, kidney, or heart tissues. This might indicate tissue-specific vulnerability of mitochondrial function to sepsis. The effect of mitochondrial transplantation on mitochondrial function as measured by high resolution respirometry was also observed only in spleen and muscle tissues. The spleen is the center of the immune system [32], and metabolism in the spleen is dramatically increased during sepsis, which might increase the vulnerability of mitochondrial function in the spleen to sepsis-induced damage.

We found the opposite course of IL-6 in spleen and plasma after 6 h in cecal slurry induced sepsis model, which has not been seen in other study. We could not know the reason, but spleen might act differently with peripheral blood leukocytes [33].

This study has several limitations. First, the specific mode of action of mitochondrial transplantation was not fully elucidated. The mechanism might be complex considering the various processes in which mitochondria are involved (e.g., energy production, redox status, apoptosis, calcium modulation, etc.), and one specific mechanism might not be responsible for the beneficial effects. Second, we did not specify the splenocytes involved in the effects of mitochondrial transplantation. T and B lymphocytes, monocytes, dendritic cells, and macrophages are all located in the spleen [33], and we could not determine which immune cells were responsible for the immunomodulatory effects of mitochondrial transplantation. Lastly, this study investigated the short-term outcome of the effect of mitochondrial transplantation in a severe abdominal infection model. However, in these days, long-term outcomes such as persistent inflammation, immune suppression and catabolism syndrome has gained much concerns, and with this investigation, we could not conclude the transplantation of mitochondria in acute phase would favor long term outcome. Despite these limitations, this study showed the beneficial effects of mitochondrial transplantation on sepsis for the first time.

## Conclusions

In cecal slurry model in rats, mitochondria transplantation increased survival, bacterial clearance, and reduced apoptosis of septic spleen, as well as alleviated inflammation by immune modulation effect of transplanted mitochondria. It has beneficial effects on sepsis and could be a promising new therapeutic approach for life-threatening sepsis cases.

## Supplementary Information

**Additional file 1.** Respiratory control rates of mitochondria in the rat sepsis model. Respiratory control rates were measured in spleen, muscle, liver and kidney tissues both before and after induction of sepsis.

**Additional file 2.** Characterization of isolated mitochondria from L6 cells and UC-MSCs.

**Additional file 3.** Distributions of transplanted mitochondria in septic spleens.

**Additional file 4.** Oxygen consumption trace in organ of cecal slurry model in rat.

**Additional file 5.** Proinflammatory cytokine expression in in vitro model of hyperinflammation and immunosuppression.

**Additional file 6.** Proinflammatory cytokine expression in in vitro model of hyperinflammation and immunosuppression with ETC inhibitors.

**Additional file 7.** Macrophage polarization in hyperinflammation model.

**Additional file 8.** Phagocytosis of E.coli-FITC in immune paralysis model.

## Data Availability

The datasets generated and analyzed during the current study are available from the corresponding author on reasonable request.

## Abbreviations

LPS: Lipopolysaccharide
RCR: Respiratory control ratio
UC-MSC: Umbilical cord mesenchymal stem cell
BSA: Bovine serum albumin
CFU: Colony forming unit
DPBS: Dulbecco’s phosphate buffered saline
TUNEL: Terminal deoxynucleotidyl transferase-mediated dUTP nick-end labeling
HRP: Horseradish peroxidase
PBMC: Peripheral blood mononuclear
RIPA: Radioimmunoprecipitation assay buffer
PVDF: Polyvinylidene difluoride
TBST: Tris buffered saline with tween20
OCT: Optimum cutting temperature
ROS: Reactive oxygen species
OXPHOS: Oxidative phosphorylation
GM-CSF: Granulocyte-macrophage colony stimulating factor
INF: Interferon
PD-1: Programmed cell death protein 1
HLA-DR: Human leukocyte antigen-DR isotype
PD-L1: Programmed death-ligand 1
MT: Mitochondria
Tol.: Tolerance
L6 cell: Rat myoblasts
DIC: Differential interference contrast
N.C: Negative control
P.C: Positive control
