# Supplementary material for: The immune modulatory effects of mitochondrial transplantation on cecal slurry model in rat
Source: Crit Care. 2021 Jan 7;25:20. doi: 10.1186/s13054-020-03436-x (PMC7789332; doi:10.1186/s13054-020-03436-x)
Supplement: Supplementary file 1 — Additional file 1. Respiratory control rates of mitochondria in the rat sepsis model. Respiratory control rates were measured in spleen, muscle, liver and kidney tissues both before and after induction of sepsis. [file 13054_2020_3436_MOESM1_ESM.docx]

**Supplementary Results**

**
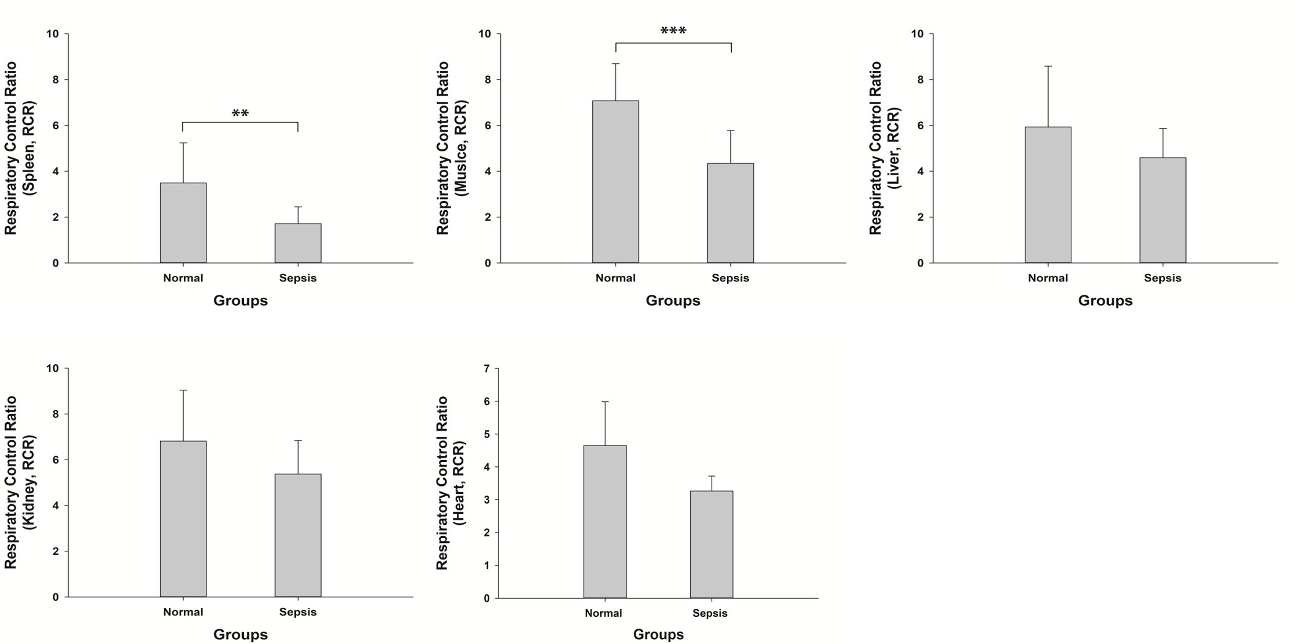
**

**Supplementary Figure S1.** Respiratory control rates of mitochondria in the rat sepsis model. Respiratory control rates were measured in spleen, muscle, liver and kidney tissues both before and after induction of sepsis. Spleen, n=9 to 14 per group; muscle, n=12 per group; liver, n=11 to 13 per group; kidneys, n=3 to 6 per group; Heart, n=2 to 6 per group. ****p* < 0 .001 and ***p* < 0 .01 compared with the normal group.
