# Supplementary material for: The immune modulatory effects of mitochondrial transplantation on cecal slurry model in rat
Source: Crit Care. 2021 Jan 7;25:20. doi: 10.1186/s13054-020-03436-x (PMC7789332; doi:10.1186/s13054-020-03436-x)
Supplement: Supplementary file 2 — Additional file 2. Characterization of isolated mitochondria from L6 cells and UC-MSCs. [file 13054_2020_3436_MOESM2_ESM.docx]

**Supplementary Results**

**
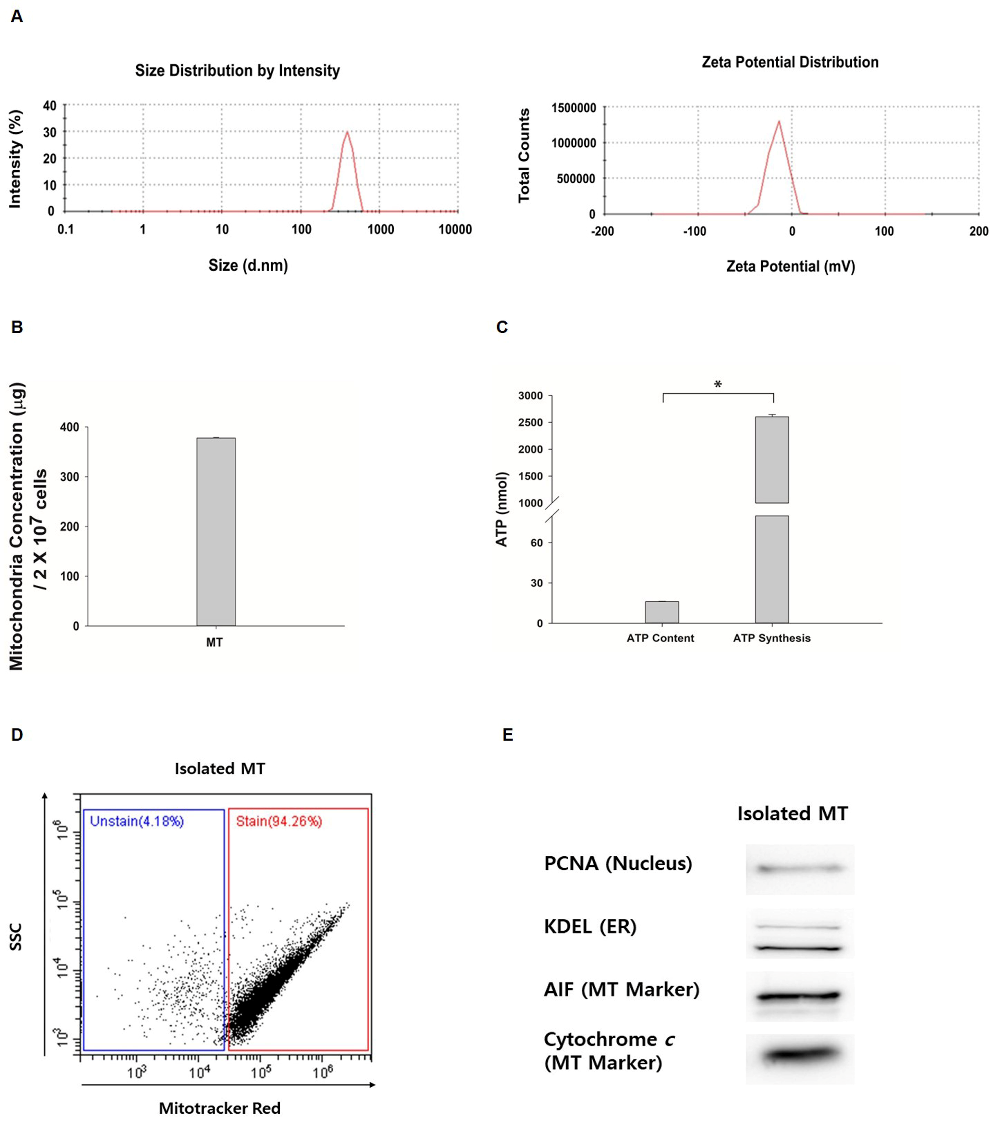
**

**Supplementary Figure S2.** Characterization of isolated mitochondria from L6 cells and UC-MSCs. (**A**) Particle size distribution and zeta potential distribution of isolated mitochondria from L6 cells (n=9). Size, μm; zeta potential, mV. (**B**) Protein concentrations in isolated mitochondria from L6 cells (n=3). (**C**) ATP content and synthesis were measured in isolated mitochondria from L6 cells. ATP content, n=6; ATP synthesis, n=3. **p* < 0.05 compared with the ATP content group. (**D**) Purity of isolated mitochondria from stained L6 cells by Mitotracker Red. (**E**) Identification of other organelles in isolated mitochondria. L6 cells, rat myoblasts; MT, mitochondria.
