# Supplementary material for: The immune modulatory effects of mitochondrial transplantation on cecal slurry model in rat
Source: Crit Care. 2021 Jan 7;25:20. doi: 10.1186/s13054-020-03436-x (PMC7789332; doi:10.1186/s13054-020-03436-x)
Supplement: Supplementary file 3 — Additional file 3. Distributions of transplanted mitochondria in septic spleens. [file 13054_2020_3436_MOESM3_ESM.docx]

**Supplementary Results**

**
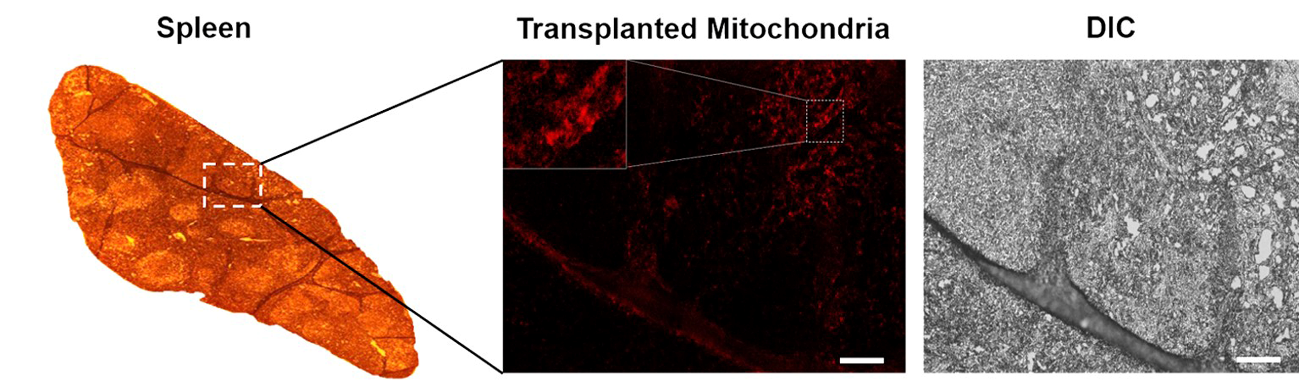
**

**Supplementary Figure S3.** Distributions of transplanted mitochondria in septic spleens. Representative histological image of a whole spleen (left). Confocal view of transplanted mitochondria (red) in the spleen (middle). DIC image of the spleen (right). DIC, differential interference contrast; N.C, negative control; P.C, positive control; MT, mitochondria.
