# Supplementary material for: The immune modulatory effects of mitochondrial transplantation on cecal slurry model in rat
Source: Crit Care. 2021 Jan 7;25:20. doi: 10.1186/s13054-020-03436-x (PMC7789332; doi:10.1186/s13054-020-03436-x)
Supplement: Supplementary file 4 — Additional file 4. Oxygen consumption trace in organ of cecal slurry model in rat. [file 13054_2020_3436_MOESM4_ESM.docx]

**Supplementary Results**

**
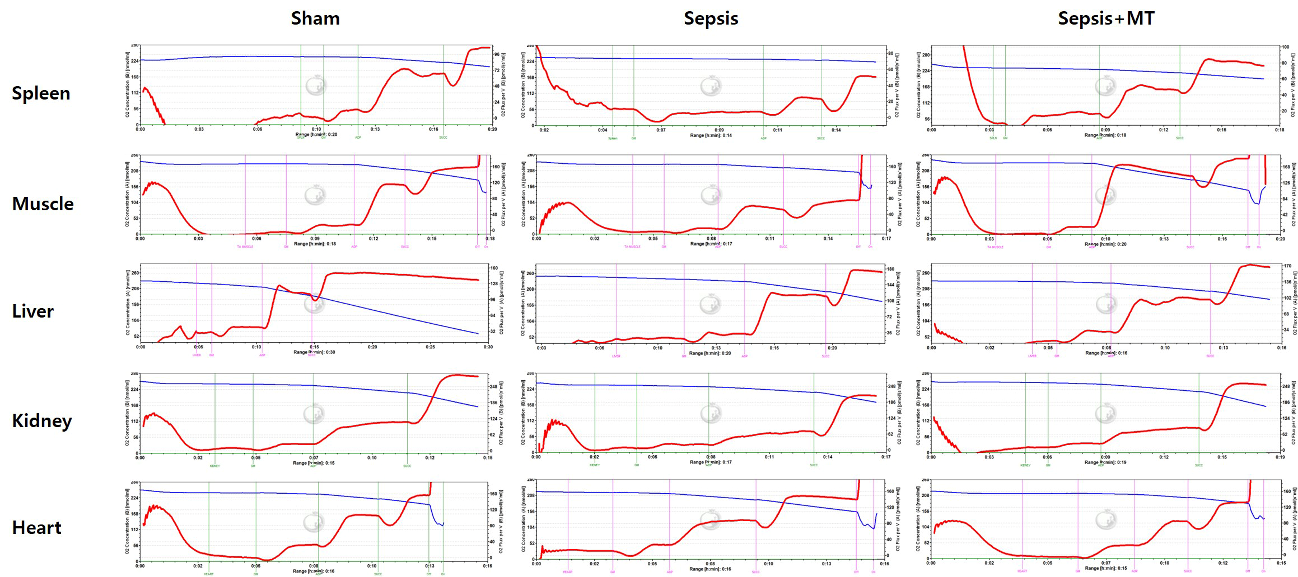
**

**Supplementary Figure S4.** Oxygen consumption trace in organ of cecal slurry model in rat. MT, mitochondria.
