# Supplementary material for: The immune modulatory effects of mitochondrial transplantation on cecal slurry model in rat
Source: Crit Care. 2021 Jan 7;25:20. doi: 10.1186/s13054-020-03436-x (PMC7789332; doi:10.1186/s13054-020-03436-x)
Supplement: Supplementary file 5 — Additional file 5. Proinflammatory cytokine expression in in vitro model of hyperinflammation and immunosuppression. [file 13054_2020_3436_MOESM5_ESM.docx]

**Supplementary Results**

**
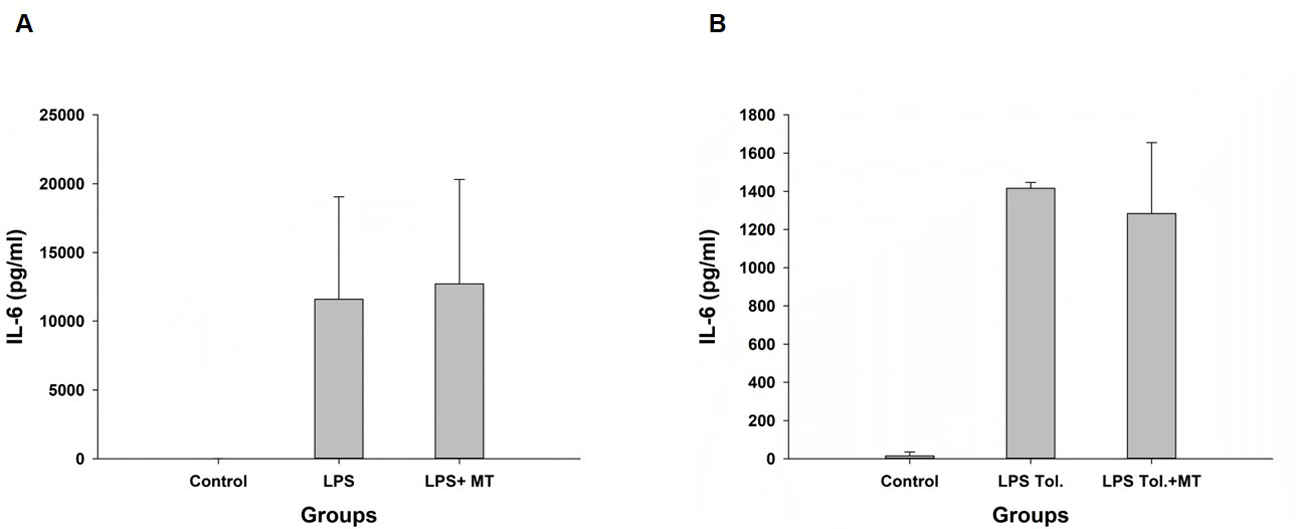
**

**Supplementary Figure S5.** Proinflammatory cytokine expression in in vitro model of hyperinflammation and immunosuppression. (**A**) Isolated monocyte from human PMBCs were stimulated with LPS and then delivered isolated healthy mitochondria. The supernatants were collected, and IL-6 expression was measured (n=2 to 3). (**B**) Isolated monocyte from human PMBCs were stimulated with LPS twice at an interval of time, and then isolated healthy mitochondria. The supernatants were collected, and IL-6 expression was measured (n=2). LPS, lipopolysaccharide; MT, mitochondria; Tol., tolerance.
