# Supplementary material for: The immune modulatory effects of mitochondrial transplantation on cecal slurry model in rat
Source: Crit Care. 2021 Jan 7;25:20. doi: 10.1186/s13054-020-03436-x (PMC7789332; doi:10.1186/s13054-020-03436-x)
Supplement: Supplementary file 6 — Additional file 6. Proinflammatory cytokine expression in in vitro model of hyperinflammation and immunosuppression with ETC inhibitors. [file 13054_2020_3436_MOESM6_ESM.docx]

**Supplementary Results**

**
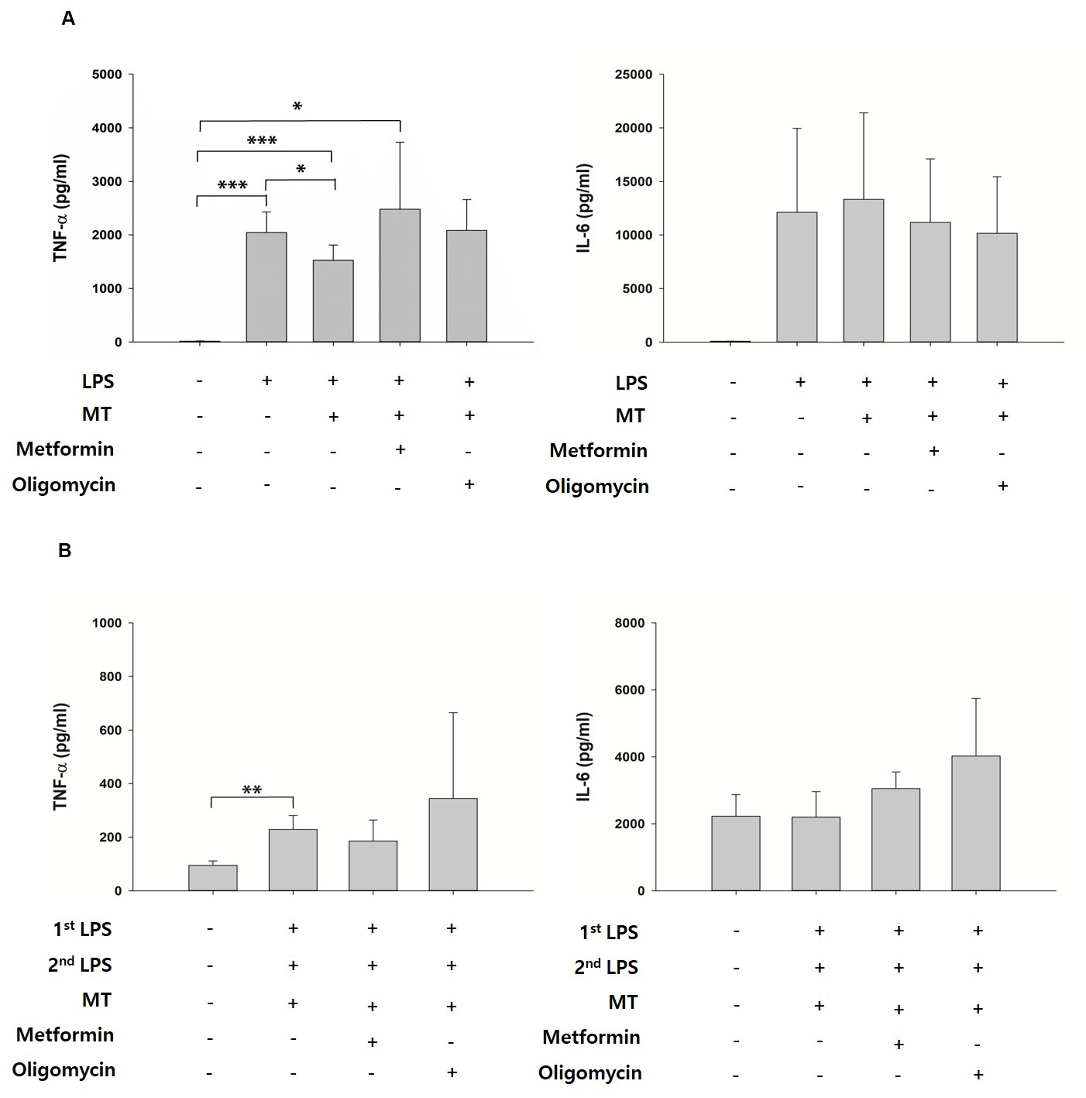
**

**Supplementary Figure S6.** Proinflammatory cytokine expression in in vitro model of hyperinflammation and immunosuppression. (**A**) Isolated monocyte from human PMBCs were stimulated with LPS and then delivered isolated healthy mitochondria and mitochondria of inhibited oxidative phosphorylation by metformin and oligomycin. The supernatants were collected, and TNF-α and IL-6 expression were measured (n=2 to 6). (**B**) Isolated monocyte from human PMBCs were stimulated with LPS twice at an interval of time, and then isolated healthy mitochondria and mitochondria of inhibited oxidative phosphorylation by metformin and oligomycin were delivered twice at and interval of time. The supernatants were collected, and TNF-α and IL-6 expression were measured (n=4 to 6). LPS, lipopolysaccharide; MT, mitochondria; Tol., tolerance. **p* < 0.05, ** *p* <0.001 and ****p* < 0.001 compared with the normal group or LPS group.
