# Supplementary material for: The immune modulatory effects of mitochondrial transplantation on cecal slurry model in rat
Source: Crit Care. 2021 Jan 7;25:20. doi: 10.1186/s13054-020-03436-x (PMC7789332; doi:10.1186/s13054-020-03436-x)
Supplement: Supplementary file 7 — Additional file 7. Macrophage polarization in hyperinflammation model. [file 13054_2020_3436_MOESM7_ESM.docx]

**Supplementary Results**

**
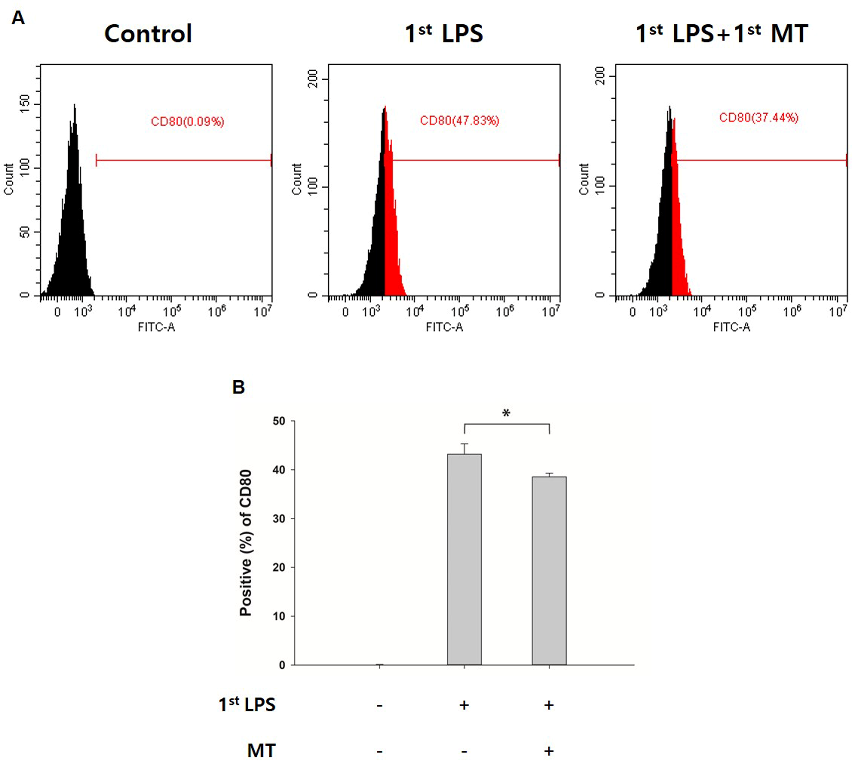
**

**Supplementary Figure S7.** Macrophage polarization in hyperinflammation model. (**A**) Representative flow cytometry histograms of CD80-FITC positive in human monocyte treated with 1^st^ LPS or 1^st^ LPS+MT (n=3). (**B**) Percentages of CD80 positive cells are presented as bar graphs (n=3). **p* < 0.05 compared with the 1^st^ LPS group. MT, mitochondria; LPS, lipopolysaccharides.
