# Supplementary material for: The immune modulatory effects of mitochondrial transplantation on cecal slurry model in rat
Source: Crit Care. 2021 Jan 7;25:20. doi: 10.1186/s13054-020-03436-x (PMC7789332; doi:10.1186/s13054-020-03436-x)
Supplement: Supplementary file 8 — Additional file 8. Phagocytosis of E.coli-FITC in immune paralysis model. [file 13054_2020_3436_MOESM8_ESM.docx]

**Supplementary Results**

**
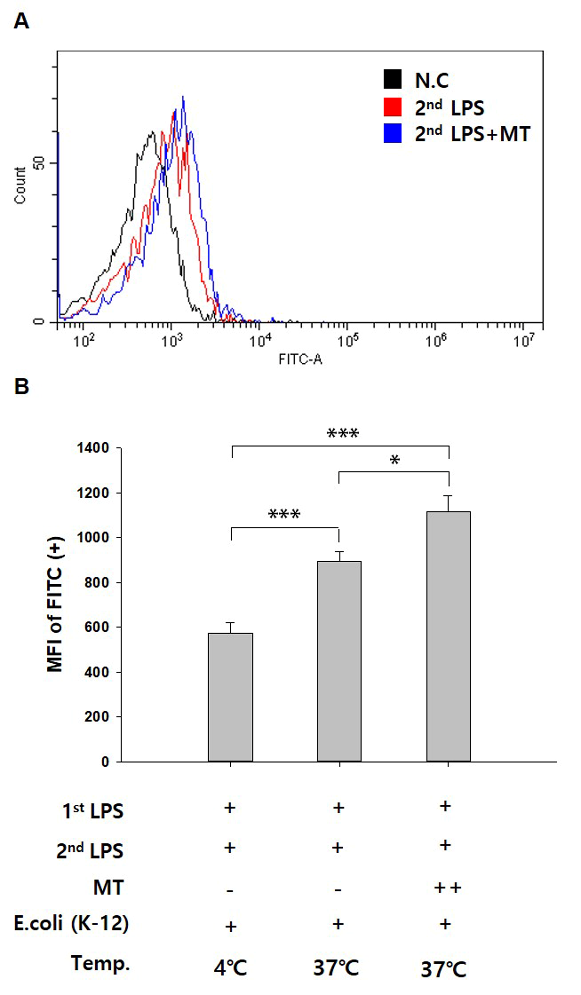
**

**Supplementary Figure S8.** Phagocytosis of E.coli-FITC in immune paralysis model. (**A**) Representative flow cytometry histograms of E.coli-FITC positive in human monocyte treated with LPS or LPS+MT twice (n=3). (**B**) Relative mean fluorescence intensity (MFI) of E.coli-FITC are presented as bar graphs (n=3). MT, mitochondria; LPS, lipopolysaccharides; N.C, negative control. **p* < 0.05 and ****p* < 0.001 compared with the N.C group or 2^nd^ LPS group.
